# A New and Sensitive HPLC-UV Method for Rapid and Simultaneous Quantification of Curcumin and D-Panthenol: Application to In Vitro Release Studies of Wound Dressings

**DOI:** 10.3390/molecules27061759

**Published:** 2022-03-08

**Authors:** Qonita Kurnia Anjani, Emilia Utomo, Juan Domínguez-Robles, Usanee Detamornrat, Ryan F. Donnelly, Eneko Larrañeta

**Affiliations:** Medical Biology Centre, School of Pharmacy, Queen’s University Belfast, 97 Lisburn Road, Belfast BT9 7BL, UK; qanjani01@qub.ac.uk (Q.K.A.); eutomo01@qub.ac.uk (E.U.); j.dominguezrobles@qub.ac.uk (J.D.-R.); u.detamornrat@qub.ac.uk (U.D.); r.donnelly@qub.ac.uk (R.F.D.)

**Keywords:** curcumin, D-panthenol, HPLC-UV, release study

## Abstract

Curcumin (CUR) and D-panthenol (DPA) have been widely investigated for wound-healing treatment. In order to analyse these two compounds from a dosage form, such as polymer-based wound dressings or creams, an analytical method that allows the quantification of both drugs simultaneously should be developed. Here, we report for the first time a validated high-performance liquid chromatographic (HPLC) method coupled with UV detection to quantify CUR and DPA based on the standards set by the International Council on Harmonization (ICH) guidelines. The separation of the analytes was performed using a C_18_ column that utilised a mobile phase consisting of 0.001% *v*/*v* phosphoric acid and methanol using a gradient method with a run time of 15 min. The method is linear for drug concentrations within the range of 0.39–12.5 μg mL^−1^ (R^2^ = 0.9999) for CUR and 0.39–25 μg mL^−1^ for DPA (R^2^ = 1). The validated method was found to be precise and accurate. Moreover, the CUR and DPA solution was found to be stable under specific storage conditions. We, therefore, suggest that the HPLC-UV method developed in this study may be very useful in screening formulations for CUR and DPA within a preclinical setting through in vitro release studies.

## 1. Introduction

Curcumin (CUR), also known as 1,7-bis(4-hydroxy-3methoxyphenol)-1,6-hepadiene-3,5-dione, is a natural compound that can be found in the rhizome of turmeric (*Curcuma longa*) [1,2]. CUR is well known for its antioxidant, anti-inflammatory and anti-infective properties [1]. Due to these properties, CUR is widely investigated in the treatment of wound healing. Several studies have reported its effectiveness as a wound-healing agent. Dai et al. (2017) found that nanofibers containing CUR exhibited a wound-healing process via fibroblast mobilisation to the wound site [3]. Additionally, another study involving in vivo topical application of CUR-based polymeric bandage showed improved healing response [1].

For the purpose of improving the wound-healing activity of CUR, an approach to combine CUR with another compound was performed in this study. D-panthenol (DPA) has the function of increasing epidermal differential in the wound-healing process [4]. Additionally, it also acts as a moisturiser by improving skin hydration and increasing water content in the skin, as well as reducing transepidermal water loss [4,5]. Upon topical application, DPA is absorbed and readily converted into pantothenic acid, which is important for the physiological function of epithelia [6]. This was proven by a previous study, where the authors reported an increase in skin hydration following 5% DPA topical administration on tattooed skin [7]. Additionally, the application of DPA on wounded human skin was able to promote the proliferation of human skin fibroblasts [8]. Due to this reason, DPA is the most promising compound to be combined with CUR to support the wound-healing process.

CUR and DPA could provide a synergetic effect in the treatment of wound healing. Accordingly, the combination of these compounds in a pharmaceutical dosage form would be beneficial and advantageous to accelerate the wound-healing process. During the development of CUR and DPA containing the dosage form, a suitable analytical method for both compounds is important to be established to support the detection and quantification of the analytes following stability and in vitro release experiments. Previously, several HPLC methods have been already used to detect and quantify CUR and DPA individually in suitable release media. For CUR, two different methods have been applied using reverse-phase (RP)-HPLC with UV detection at 425 nm, which obtained LOQ values of 2.73 μg/mL [9] and 0.5 μg/mL [10]. Additionally, RP-HPLC methods were also applied for DPA detection and quantification at 205 nm with LOQ values of 6.2 μg/mL [11] and 13.72 μg/mL [12].

There has been no published work developing a simultaneous quantification method for CUR and DPA. Accordingly, in this paper, for the first time, we develop a simultaneous method to analyse CUR and DPA using HPLC. There is quite a challenge to detect both of these compounds simultaneously considering their different physicochemical properties, such as water solubility and maximum UV absorbance. This work started with the development of a simultaneous quantification method of CUR and DPA. Subsequently, the method was validated according to ICH and applied for stability and in vitro release studies using suitable release media.

## 2. Materials and Methods

### 2.1. Materials

CUR (purity 95%) and DPA (purity > 98%) were purchased from Alfa Aesar (Lancashire, UK). Two different poly(caprolactone) (PCL), PCL 6506 (MW = 50,000 g/mol, i.e., high molecular weight), henceforth referred to as H-PCL, and PCL 2054 (MW = 550 g/mol, i.e., low molecular weight), henceforth referred to as L-PCL, were donated by Perstorp (Malmö, Sweden). Ultrapure water was obtained from a water purification system Elga PURELAB DV 25 (Veolia Water Systems, Ireland). HPLC column XSelect CSH C_18_ (Waters, 3.0 × 150 mm, 3.5 μm particle size) were purchased from Waters (Dublin, Ireland). All other chemical reagents were of analytical grade and purchased from Sigma-Aldrich (Dorset, UK) and Fisher Scientific (Leicestershire, UK).

### 2.2. Preparation of Stock, Calibration Standards and Quality Control Solution

Stock solutions of CUR and DPA were separately prepared by dissolving 10 mg of each drug in methanol and PBS (pH 7.4) in a 10 mL volumetric flask to achieve a 1 mg mL^−1^ final concentration for both compounds. The working solutions were diluted into serial concentrations for standard calibration, as well as for the quality control (QC) solutions. Calibration standards (CSs) were obtained by diluting the stock solution of both drugs in a volumetric flask to obtain a range of concentrations from 0.19 to 12.5 µg mL^−1^ for CUR and from 0.19 to 25 µg mL^−1^ for DPA. QC solutions were also prepared at three different concentrations in six replicates. Concentrations of QC solutions used in this study were 10, 2 and 0.75 µg mL^−1^ for CUR and 20, 5 and 0.75 µg mL^−1^ for DPA. All QC solutions were prepared in PBS (pH 7.4) containing 0.5% *w*/*v* Tween^®^ 80.

### 2.3. Instrumentation and Chromatographic Conditions

Simultaneous analysis of CUR and DPA was conducted by utilising an HPLC system consisting of an Agilent Technologies 1220 Infinity compacted LC series (Agilent Technologies UK Ltd., Stockport, UK) with a degasser, binary pump, standard autoinjector and UV detector. All analyte separation was carried out on an XSelect CSH C_18_ column (Waters, 3.0 × 150 mm) with a particle size of 3.5 μm and a pore size of 130 Å, fitted with a VanGuard^®^ cartridge (Waters, 3.9 × 5 mm). The mobile phase consisted of a mixture of 0.001% *v*/*v* of phosphoric acid in water (mobile phase A) and acetonitrile (ACN) (mobile phase B). The analysis was performed at room temperature for 15 min using the gradient condition, as detailed in Table 1. The injection volume of all samples was 20 µL, and the flow rate was 0.8 mL min^−1^.

### 2.4. HPLC Method Validation

#### 2.4.1. Linearity, LOD, and LOQ

Linearity was assessed by creating the calibration curve derived from the CSs mentioned previously in Section 2.2. The calibration curves were obtained by analysing seven CSs containing different CUR and DPA concentrations. The limit of detection (*LOD*) and limit of quantification (*LOQ*) were determined using the following equations.

(1) $$LOD=\frac{3.3\;\sigma }{S}$$

(2) $$LOQ=\frac{10\;\sigma }{S}$$

where *σ* is the standard deviation (*SD*) of the responses of the calibration curve, and *S* is the slope of the calibration curve.

#### 2.4.2. Accuracy and Precision

Accuracy and precision of the method were determined at three different concentrations (low, medium, and high) in six replicates for each QC sample. Accuracy and precision were evaluated intraday and interday by calculating the relative standard deviation (*RSD*) of the responses for determining accuracy and calculating the relative errors (*RE*) for evaluating precision. The method was deemed to be accurate and precise if the *RSD* and *RE* from all samples fell below 15% [13]. Both *RSD* and *RE* were calculated using the following equations.

(3) $$RSD\;\left(\%\right)=\frac{SD}{Mean\;of\;concentration}\times 100\%$$

(4) $$RE\;\left(\%\right)=\frac{Absolute\;error}{True\;value}\times 100\%$$

where *Absolute error* is the value determined from deviation between the measured concentration and the theoretical concentration of the standard (*True value*).

### 2.5. Stability Studies

Stability studies of CUR and DPA were conducted to assess the degradation of the stock solution in the presence of ascorbic acid in the release media in different conditions, which represent the conditions of in vitro release study and storage conditions. A stock solution of 1 mg mL^−1^ for both drugs was prepared, diluted with an appropriate volume of PBS (pH 7.4) containing 0.5% *w*/*v* Tween^®^ 80 or PBS (pH 7.4) containing 0.5% *w*/*v* Tween^®^ 80 and 0.1% *w*/*v* of ascorbic acid (AA), and placed in a glass vial with a final concentration of 5 µg/mL. The solutions were kept at room temperature, in the fridge (2–8 °C), in an incubator at 37 °C and in the freezer at −20 °C. All glass vials were covered with aluminium foil to avoid any light exposure during the study. At a predetermined time, 1 mL samples were taken from each vial over 30 days. All samples were prepared in triplicate and analysed using the validated method of HPLC to evaluate the percentage of recovery.

### 2.6. Preparation of CUR-DPA Meshes for Wound-Healing Dressings Using a Robocasting 3D Printer

PCL-based meshes with a diameter of 1.3 cm were initially designed using computer-aided (CAD) software and subsequently printed using a robocasting 3D printer (Bioscaffolder 3.2., GeSiM) (Radeberg, Germany). Prior to the printing process, 5% *w*/*w* CUR and 5% *w*/*w* DPA were mixed with H-PCL/L-PCL (with the ratio of 60%/40%) using SpeedMixer^TM^ DAC 150.1 FVZ-K at 3500 rpm for 5 min. The mixture was subsequently placed into a metal syringe equipped with a 0.5 mm nozzle. The print speed and temperature were set at 10 mm/s and 60 °C, respectively. The layer height was 0.25 mm, and the strand distance was 1.6 mm. The angle of the layer was changed to 90° for the second layer in order to shape a mesh. The surface morphology of the drug-loaded 3D-printed meshes was evaluated by using scanning electron microscopy (SEM) (Hitachi TM3030; Tokyo, Japan) and a Leica EZ4 D digital microscope (Leica, Wetzlar, Germany).

### 2.7. Application of the Method to In Vitro Release Study

In order to study the release profiles of CUR and DPA from the PCL-based meshes, an in vitro release experiment was set up using PBS (pH 7.4) with the addition of 0.5% *w*/*v* Tween^®^ 80 and 0.1% *w*/*v* AA as the release media. An individual mesh was placed in a glass bottle containing 50 mL of release media to maintain sink condition. This experiment was performed at 37 °C and shaken at 40 rpm. At the predetermined time, the mesh was removed, dried, and placed into a new bottle containing 50 mL of fresh release media. The previous release media was then collected and analysed using the validated simultaneous analytical method of CUR and DPA (*n* = 3).

### 2.8. Statistical Analysis

Least-squares linear regression analysis, %RSD, %RE, LOD, LOQ and calculation of means and SD were all performed using Microsoft^®^ Excel 2019 (Microsoft Corporation, Redmond, WA, USA). Additionally, statistical analysis of drug stability studies was carried out using GraphPad Prism^®^ version 9.3.1 (GraphPad Software Inc., San Diego, CA, USA). Two-way ANOVA was used for comparison using two factors, namely storage conditions and storage duration. In all cases, *p* < 0.05 was used to denote statistical significance.

## 3. Results and Discussion

### 3.1. Chromatographic Method Development

The RP-HPLC method with a gradient was implemented in this study. Different UV wavelengths were used in the analytical method due to the different UV absorbances of CUR and DPA. CUR is present in yellowish solution due to long intense wavelength absorption in the visible region [14]; therefore, it was analysed at 425 nm. On the other hand, DPA is a colourless liquid and does not exhibit strong UV–visible absorption; therefore, it must be quantified by using a low UV wavelength [12,15]. Accordingly, we analysed DPA at 200 nm.

**Figure 1:**
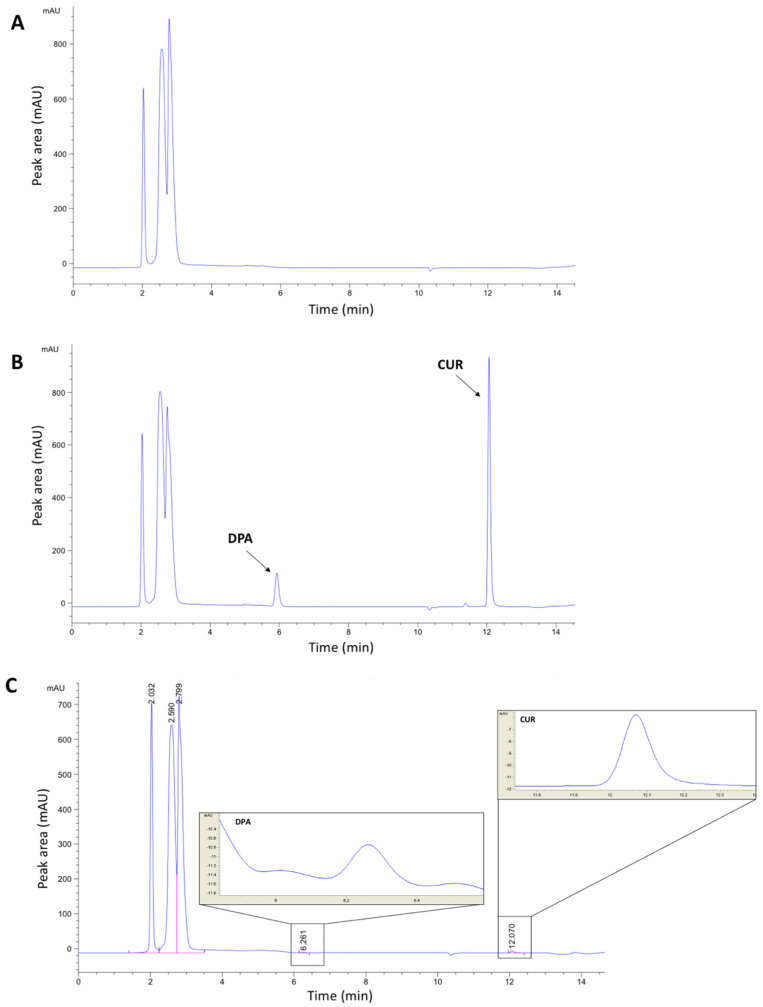
Representative chromatograms of CUR and DPA in release media: (**A**) blank release media (PBS pH 7.4 containing 0.5% *w*/*v* Tween^®^ 80 and 0.1% *w*/*v* of ascorbic acid); (**B**) CUR and DPA (12.5 µg mL^−1^ for both drugs) in release media; (**C**) LOD chromatogram (0.019 µg mL^−1^).

In this study, sample analysis was carried out on an RP C_18_ column due to its versatility and suitability for a broad range of compounds [16]. Therefore, this system was beneficial to our study, specifically in the separation of CUR and DPA as nonpolar and polar compounds, respectively, as shown in Figure 1. Additionally, the molecules of each compound are able to present different ionisation states depending on pH values. Accordingly, the composition of the mobile phase was optimised to obtain a consistent degree of ionisation and polarity of the analytes. CUR and DPA are fully converted to ionised forms at a pH range between 1.0 and 5.8 for CUR and 1.0 and 10.2 for DPA [17]. Accordingly, 0.001% *v*/*v* of phosphoric acid in water with a pH of 3.10 was used in this study. This pH was considered to be a suitable condition in order to achieve full ionisation of both CUR and DPA in a constant manner.

### 3.2. HPLC Method Validation

#### 3.2.1. Linearity, LOD, and LOQ

The LOD and LOQ of this method and the characteristics of the calibration curve obtained, including the slope, y-intercept and coefficient of correlation, are presented in Table 2. The results suggest that the calibration curves for both drugs exhibited a linear response with the regression coefficient (R^2^) ≥ 0.998 over the concentration range analysed [16,18,19].

This is the first report of an HPLC method for the determination of DPA and CUR together. Some previous studies have reported HPLC methods to determine DPA or CUR independently. However, there are some previously published works describing the simultaneous determination of DPA and other compounds such as lidocaine, mepyramine or resorcinol in different formulations [12,20]. These methods presented higher LOD and LOQ values than the ones reported in this work. In all cases, the LOD was higher than 1 μg/mL [11] for all the analysed compounds. Independent methods for the determination of DPA have been reported. Kulikov et al. developed a method to determine DPA in pharmaceutical formulations using reverse-phase HPLC. The method presented an LOD of 2.1 and LOQ of 6.2 μg/mL for the single detection of DPA [11]. Alternatively, there are a wide variety of methods described in the literature for CUR determination. Several methods have been described for the simultaneous determination of CUR and other derivatives or antioxidant compounds such as quercetin [21,22,23].

#### 3.2.2. Accuracy and Precision

The results of accuracy and precision for the QC samples are reported in Table 3. The analytical method for simultaneous CUR and DPA quantification was accurate, since the values of %RE of all QC samples were found to be within the range of 0.56 to 8.74%. These values are acceptable, as they fall within the required limits of 15%. In terms of precision, %RSD values were found within the range of 1.00 to 5.54%. These values are acceptable based on the recommendations of González et al. [13] that both values of %RE and %RSD should not exceed 15%. In addition, the changes in temperature and instrument conditions did not significantly affect the developed method, as shown in Table S1, indicating the method is accurate, precise and reliable.

### 3.3. Stability Study

**Figure 2:**
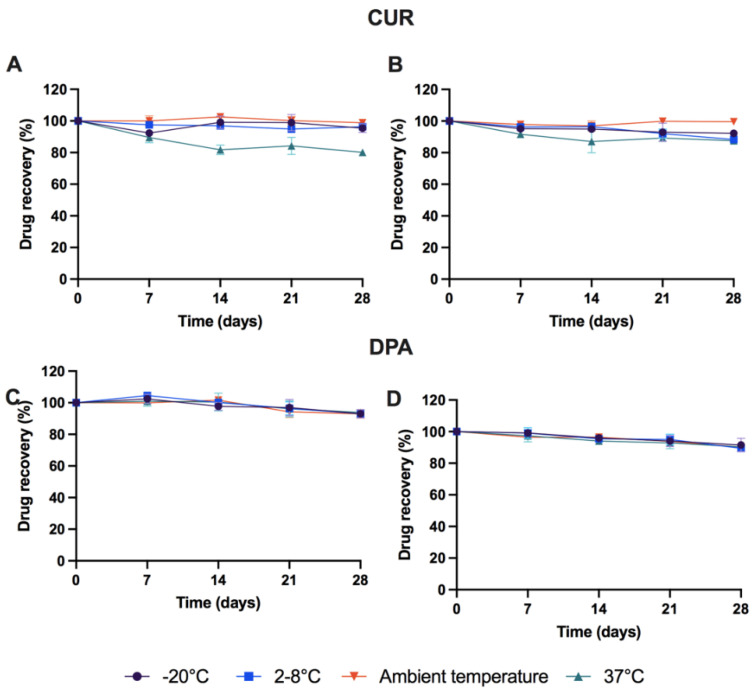
Percentage recovery of CUR: (**A**) PBS pH 7.4 containing 0.5% *w*/*v* Tween^®^ 80; (**B**) PBS pH 7.4 containing 0.5% *w*/*v* Tween^®^ 80 and 0.1% *w*/*v* AA; DPA in (**C**) PBS pH 7.4 containing 0.5% *w*/*v* Tween^®^ 80; (**D**) PBS pH 7.4 containing 0.5% *w*/*v* Tween^®^ 80 and 0.1% *w*/*v* AA after different conditions over 30 days (means ± SD, *n* = 3).

Stability of CUR-DPA solution within release media was investigated. In this study, several conditions were evaluated to establish suitable sample storage conditions. Additionally, the presence of AA was investigated as an antioxidant and potential stability enhancer for CUR-DPA solution in the release media. As shown in Figure 2, DPA is considered to be relatively stable under the different conditions tested over 28 days. The addition of AA to the samples did not affect DPA concentrations (*p* > 0.05). Moreover, CUR required AA to be stored at higher temperatures (37 °C), as the amount of CUR remaining after 28 days decreased to 80% in the absence of AA. This result suggests that storage temperature could affect the stability of CUR, which confirms the findings of another study reporting the degradation of CUR at high temperatures [24,25,26]. On the other hand, CUR was stable for at least 30 days under the conditions studied in this work when AA was added to the release media. Accordingly, these results indicate that there was a need to use AA as an antioxidant in the in vitro release studies. These results are in line with previously published research [25,26,27]. Moreover, in order to avoid any degradation from CUR and DPA during the study, samples obtained could be stored in the −20 °C freezer for 28 days prior to HPLC analysis.

### 3.4. Preparation of CUR-DPA Topical Meshes Using a Robocasting 3D Printer

CUR and DPA have been both reported to improve wound healing [2,28]. DPA acts as a moisturiser and skin barrier enhancer [4,5]. On the other hand, CUR takes part in several stages of the wound-healing process, accelerating it [1]. Accordingly, the combination of these two compounds can provide noticeable enhancement for wound care. The combination of these two compounds for the manufacturing of wound dressings has never been described before. These two compounds have been combined using 3D-printing technology to prepare a novel dosage form. The use of this technology has been gaining momentum in recent years for the development of topical dosage forms [6,29,30].

**Figure 3:**
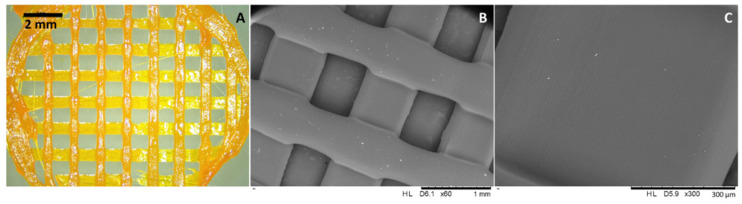
Digital microscopy (**A**) and SEM (**B**,**C**) images with different magnification of the drug-loaded 3D-printed meshes.

PCL-based topical meshes were successfully prepared using a robocasting 3D printer. The presence of CUR within the meshes could be confirmed by the bright orange colour of the resulting meshes (Figure 3A). It can be inferred that the drug was successfully combined with the PCL matrix. This was corroborated by using SEM (Figure 3). SEM pictures of the drug-loaded meshes presented a smooth surface, and no drug aggregates were observed on their surface (Figure 3B,C). This suggests that the premixing process, using a dual asymmetric centrifugal laboratory mixer system and the 3D printing process itself, were successfully applied. This part of the work showed the versatility and simplicity of 3D printing technology in the preparation of pharmaceutical dosage forms. Moreover, this technology can be applied to prepare personalised implantable drug delivery devices in various shapes and sizes [31,32,33,34]. In terms of the composition of the resulting meshes, the combination of H-PCL and L-PCL was used as the main polymer due to its good homogeneity and printability properties, which has been proven by previous studies [35,36,37]. Additionally, L-PCL allowed drug dissolution within the thermoplastic H-PCL matrix without using any potentially harmful solvents as required by using other manufacturing processes, such as electrospinning. It can be clearly seen that the prepared CUR and DPA loaded meshes possessed good homogeneity and acceptable shape and size.

### 3.5. Application of the Method to In Vitro Release Study

**Figure 4:**
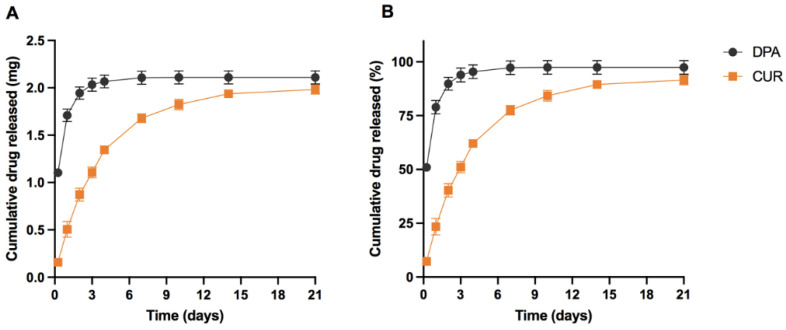
Release profiles of CUR and DPA in PBS (pH 7.4) with the addition of 0.5% *w*/*v* of Tween^®^ 80 and 0.1% *w*/*v* of ascorbic acid over 21 days. Cumulative drug released (**A**) in mg and (**B**) in percentage. Data points are expressed as means ± SEM., *n* = 3.

The amount of CUR and DPA released from the meshes was quantified using the validated analytical method. The release profiles of CUR and DPA are shown in Figure 4. In general, the release of CUR and DPA can be sustained for up to 21 days. Interestingly, CUR and DPA present different release rates. The release rate of CUR was slower than DPA. At the beginning, the amount of CUR released was fivefold lower than the amount of DPA released. After 4 days, the meshes were able to release around 2 mg of DPA and slowly increased until Day 21. On the other hand, 2 mg of CUR were released after 21 days. This different phenomenon could be explained by the different solubility of CUR and DPA in water. DPA is a water-soluble compound [38], while CUR is practically insoluble in water at acidic and neutral pH values [39]. Considering the hydrophobic nature of CUR, Tween^®^ 80 was added to the release media to maintain sink conditions for CUR release [29,40,41]. Despite using a hydrophobic PCL-based matrix, the solubility of the drugs played an important role in drug release kinetics. This release experiment shows the utility of a method capable of simultaneously determining DPA and CUR. The use of HPLC methods capable of determining more than one drug simultaneously has the potential to save analysis time and solvents.

## 4. Conclusions

The current work highlights the development and validation of a simultaneous HPLC method for the sensitive and reliable detection and quantification of CUR and DPA. Both drugs have been widely studied for wound-healing treatment and could provide a synergistic effect in the management of a wound. The developed method was successfully validated according to ICH guidelines. The simultaneous method was found to be linear, accurate and precise over a range of concentrations for each drug. In addition, the solution containing both CUR and DPA was found to be stable under the specific storage condition over 28 days. The HPLC-UV method was successfully applied to determine both CUR and DPA release profiles from topical mesh formulations in in vitro release study. It is hoped that this method may be of great heuristic value in preclinical CUR and DPA formulation screening, evaluation and development.

## Figures and Tables

**Table 1:** Gradient conditions for simultaneous analysis of CUR and DPA.

Time (min)	A (%)	B (%)	Detector Wavelength (nm)
0–7	94	6	200
7–12	25	75	425
12–15	94	6	200

**Table 2:** Properties of the calibration curve for quantification of CUR and DPA with LOD and LOQ values (*n* = 6).

	Analyte	Concentration Range (µg mL^−1^)	Slope	*y*-Intercept	R^2^	LOD (µg mL^−1^)	LOQ (µg mL^−1^)	RSS
**Intra-day**	CUR	0.39–12.5	226.63	−20.81	0.9999	0.09	0.29	673.35
	DPA	0.39–25	40.69	−0.19	1	0.08	0.24	28.08
**Inter-day**	CUR	0.39–12.5	209.98	−24.258	1	0.12	0.36	809.11
	DPA	0.39–25	40.93	−1.32	1	0.10	0.31	14.72

**Table 3:** Intraday and interday accuracy and precision of CUR and DPA in PBS (pH 7.4) (means ± SD, *n* = 6).

Analyte	Concentration Added (µg mL^−1^)	Intraday			Interday		
		Concentration Found (µg mL^−1^)	Precision (%RSD)	Accuracy (%RE)	Concentration Found (µg mL^−1^)	Precision (%RSD)	Accuracy (%RE)
CUR	10	10.09	2.79	0.91	10.16	1.01	1.58
	2	1.83	2.20	−8.74	1.89	5.54	−5.75
	0.75	0.77	2.30	3.32	0.75	1.16	0.56
DPA	20	19.44	1.00	−2.82	21.39	2.54	6.93
	5	4.88	1.77	−2.36	5.11	2.71	2.15
	0.74	0.75	2.90	0.96	0.73	5.27	−1.91

## Data Availability

Not applicable.

## Supplementary Materials

The following supporting information can be downloaded at: https://www.mdpi.com/article/10.3390/molecules27061759/s1, Table S1: Reliability testing of the analytical method using two different conditions (means ± %CV, *n* = 3).
