# Supplementary material for: A New and Sensitive HPLC-UV Method for Rapid and Simultaneous Quantification of Curcumin and D-Panthenol: Application to In Vitro Release Studies of Wound Dressings
Source: Molecules. 2022 Mar 8;27(6):1759. doi: 10.3390/molecules27061759 (PMC8954134; doi:10.3390/molecules27061759)
Supplement: Supplementary file 1 [file molecules-27-01759-s001.zip › molecules-1612992-supplementary.pdf]

A new and sensitive HPLC-UV method for rapid and simultaneous quantification of curcumin and D-panthenol: Application to *in vitro* release studies of wound dressings

Qonita Kurnia Anjani[1], Emilia Utomo[1], Juan Domínguez-Robles[1], Usanee Detamornrat[1], Ryan F. Donnelly[1], Eneko Larrañeta[1]*

[1]School of Pharmacy, Queen's University Belfast, Medical Biology Centre, 97 Lisburn Road, Belfast BT9 7BL, UK

*Correspondence: e.larraneta@qub.ac.uk; School of Pharmacy, Queen's University Belfast, Medical Biology Centre, 97 Lisburn Road, Belfast BT9 7BL, Northern Ireland, UK

The application of the developed method using different HPLC apparatus at different temperatures (room temperature and 30°C) has been conducted to investigate the reliability of the method (Table S1). Accordingly, no significant differences were found in the retention time and peak area results of samples containing DPA and CUR using 2 different conditions ($p > 0.05$). Therefore, these results are indicating that the validated method was accurate, precise, and reliable. Both, HPLC A and B, are the same type of An Agilent technologies 1220 infinity compacted LC series consisting of Agilent degasser, binary pump, auto standard injector and detector (Agilent Technologies UK Ltd, Stockport, UK). However, HPLC A is not equipped with temperature controller such as HPLC B.

Table S1. Reliability testing of the analytical method using two different conditions (means ± %CV, n = 3).

Condition		DPA		CUR	
	Concentration ($\mu g\ mL^{-1}$)	Retention time (min)	Peak area (mAU)	Retention time (min)	Peak area (mAU)
HPLC A, room temperature	12.5	6.09 ± 1.09	507.90 ± 1.14	12.05 ± 0.23	5235.10 ± 1.75
	6.25	6.09 ± 0.97	250.70 ± 2.51	12.06 ± 0.26	2590.43 ± 1.71
	3.125	6.08 ± 0.90	125.80 ± 4.17	12.05 ± 0.26	1266.53 ± 1.34
HPLC B, Column temperature at 30°C	12.5	6.49 ± 0.73	506.73 ± 1.15	12.12 ± 1.33	5575.70 ± 0.06
	6.25	6.48 ± 0.19	249.77 ± 0.86	12.13 ± 2.02	2795.83 ± 0.04
	3.125	6.41 ± 0.75	123.91 ± 0.88	12.13 ± 6.53	1378.00 ± 0.07
